# The Influence of Family Multi-Institutional Involvement on Children’s Health Management Practices

**DOI:** 10.3390/children9060828

**Published:** 2022-06-03

**Authors:** Leslie Paik

**Affiliations:** School of Social and Family Dynamics, Arizona State University, Tempe, AZ 85287, USA; leslie.paik@asu.edu

**Keywords:** family involvement, adolescent health, chronic illness, cultural health capital

## Abstract

Given the increasing prevalence of youths with chronic medical conditions and the racial, gender, and class disparities in health in the U.S., it is important to understand how families manage their youths’ health condition during the transitional time of adolescence when parents and youths are renegotiating their respective roles and responsibilities related to that condition. This paper explores a relatively understudied factor to this fraught and often confusing process: family involvement in multiple institutions for both health and non-health related issues. Based on qualitative fieldwork with 33 families in New York City whose youths have chronic health conditions (e.g., diabetes, asthma, obesity), the paper shows how family multi-institutional involvement can sap family resources in often unexpected ways. This type of institutional involvement has greater implications for poor and minority families who are more likely to be compelled to participate in these organizations with less influence to shape their cases as opposed to middle class and white families. In sum, this paper provides a more nuanced perspective of parental involvement in youths’ health management practices as a fluid evolving process shaped in part by family involvement in other institutions.

## 1. Introduction

Health practitioners view adolescence as the time for youths to take ownership of managing their chronic health-related conditions as they transition from pediatric to adult medical care, while the parents who used to be more in control of that management must learn to cede that authority to their youths. Yet, parents are still legally and morally responsible for the youths and as it pertains to the youth’s health, remain involved even if only in practical terms (e.g., obtaining and paying for medication, buying food for the family, taking youths to appointments). These competing messages can be confusing as to who is expected to do what, potentially leading to conflict between youth, parents, and medical staff and affecting the youth’s health. Given the increasing prevalence of children with chronic medical conditions and the racial, gender, and class disparities in health in the U.S., it is important to understand more about how families view and manage youths’ health conditions during this transitional time when parents and youths are renegotiating their respective roles and responsibilities related to the condition.

This paper seeks to do just that, exploring a relatively understudied factor to this fraught time: family involvement in multiple institutions for both health and non-health related issues. Based on qualitative fieldwork of 94 interviews and 100 home visits with 33 families in New York City whose adolescent youths have chronic health conditions (e.g., diabetes, asthma, obesity), I explore the following interrelated research questions: How is parents’ involvement in their youth’s health management influenced by their involvement with other family members’ health issues or in other institutions (e.g., schools, courts, housing, welfare)? How does this multi-institutional involvement shape parents’ and youths’ views about their responsibilities in managing the latter’s health during this transitional time of adolescence? In addressing those questions, the paper shows how this type of multi-institutional involvement can sap family resources in often unexpected ways. As I have discussed elsewhere [1], multi-institutional involvement has implications for poor and minority families, as they are often more likely to be compelled to engage with these institutions with less influence to shape their cases as opposed to middle class and white families who have more options to seek services in private institutions. Fernandez-Kelly [2] characterizes this phenomenon as one of “distorted engagement,” in which poor and minority families are subjected to greater scrutiny in public “liminal institutions” that offer lesser quality services while middle- and upper-class families can opt to turn to private institutions if they deem fit. These inequalities across institutions further complicate the family’s role in managing adolescent health, potentially worsening health inequalities.

By studying families’ management of their youth’s illness in the context of their multi-institutional involvement, this paper expands on conceptualizations of family-centered health approaches, particularly for families who face other challenges associated with racism and poverty [3]. Much of the previous research in this area has looked at two facets: (1) illness work, or what families do to manage the medical aspects of the disease and the effect of the illness on a person’s self-concept in the context of their everyday lives [4,5,6,7,8,9,10,11,12,13,14,15,16,17,18,19,20], and (2) cultural health capital, or the family’s knowledge and practices related to the illness, that informs the family’s interactions with medical professionals [21,22,23]. While this research points to different ways that economic resources can shape family life and youths’ health, much of it has largely been related to families’ actions related to one ill member. The analytical question remains how family involvement in youths’ health management might work differently or in more complex ways if multiple institutions are providing treatment or if the family must navigate different institutions for many members’ illnesses and other issues. Moreover, it is unclear if and how the influence of cultural health capital changes when a parent deals with multiple illnesses at once or how doctors’ perceptions of parents’ cultural health capital might be affected if they do not have a grasp of the scope of institutional involvements within the household. While others [21,24,25] have discussed families’ experiences with multiple members’ illnesses, they do not look at family institutional involvement beyond simply medical ones to show how they affect the youths’ health management practices. In response, this paper argues that we need to expand our analytical focus to consider family involvement in multiple institutions that could be related just to the youths’ illnesses as well as for other family members within and across households. In doing so, it provides a more nuanced understanding of how institutions shape family life, their economic resources, and the youth’s’ health management practices.

## 2. Materials and Methods

The data for this paper comes from a qualitative study of families in one borough in New York City, the Bronx, which has a high prevalence of chronic medical conditions such as diabetes, obesity, and asthma. According to the 2021 County Health Rankings and Roadmaps [26], the Bronx came in last in overall health outcomes in the state, or 62nd out of 62 counties; the specific outcomes were premature death (years of potential life lost), low birthweight, and three self-reported measures (poor or fair health, poor physical health days, poor mental health days). The prevalence of diabetes and obesity for adults aged 20 and older in the Bronx was 13% and 29%, respectively, compared to 10% and 26% in New York state [26]. The Bronx also has the highest rates of asthma in the city: according to the New York City Department of Health and Mental Hygiene’s Environmental and Health Data Portal [27], in 2017, 17.5% of children in the Bronx aged 0–13 years old were diagnosed with asthma, compared to 11.2% of children citywide; 6.8% of adults had asthma in the last 12 months, compared to 4.3% citywide. In addition, the rates of asthma-related emergency room visits for children (ages 5–17 years old) in the Bronx were 410 per 10,000 compared to 223 per 10,000 citywide in 2018.

**Figure 1:**
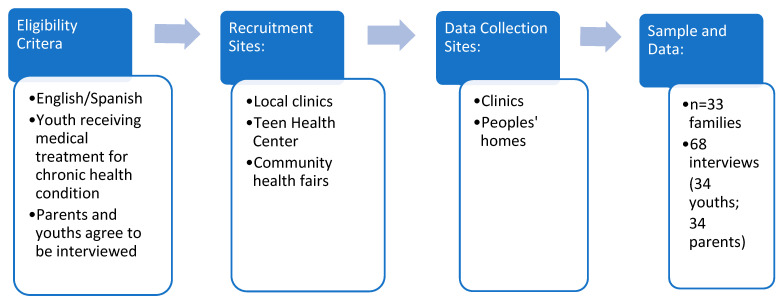
Phase 1 research design and data collection.

After receiving IRB approval for the study, I started the first phase of the study in 2015, depicted below in Figure 1. I recruited families through pediatric and adolescent clinics run by a private New York City hospital. My research assistants and I approached families in the waiting room, or pediatricians referred them to us. Fliers for the study were also posted in the waiting room and provided to staff to hand out to families. In addition, the hospital had a teen health center that conducted community outreach; my research assistants and I joined them at health fairs to recruit families. These one-time interviews were conducted in English and Spanish between 2015–2016. My research assistants and I followed an interview protocol of open-ended questions to ensure consistency among the data. These questions focused on families’ strategies in managing the youths’ illness and their experiences with doctors and health care institutions related to those illnesses. Of concern for this paper, many families mentioned other sick members besides the youth and several institutional involvements. The interviews ranged from 25 min to 1.5 h, with parents’ interviews lasting longer than the interviews with youths. In total, 68 initial interviews (34 parents and 34 youths in 33 families) were completed. 

**Figure 2:**
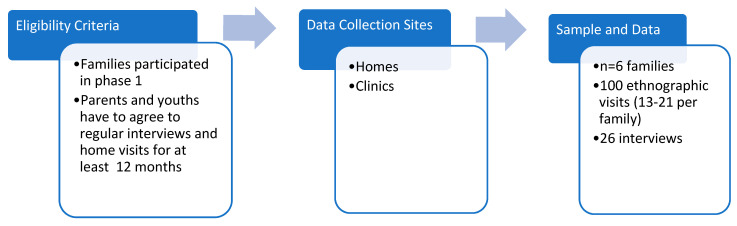
Phase 2 research design and data collection.

In the second phase of the study conducted between 2015 and 2017 depicted in Figure 2, I did an ethnography of six families from the interview study to get a better sense of how they managed the youths’ illness within the context of their everyday life. This ethnography mainly consisted of home observations over at least a 12-month period for each family; whenever possible, I accompanied the family to the youths’ medical appointments to see how they interacted with the doctors and other staff. In total, I did 100 visits, ranging from 13–21 visits per family. The visits lasted between one to four hours where I spent time talking with the families and observed how they interacted with one another. To build rapport with the families, I did not take down jottings during the visit. Rather I took down jottings immediately after the visits and wrote up more detailed fieldnotes within one or two days of the visits. In addition, I conducted two to three follow-up interviews every three to four months with the parents and youths. These interviews focused on any recent medical issues that had arisen since the first interview, as well as other issues that were going on in the families’ lives at that moment. I conducted 26 follow-up interviews with the families for a total of 94 interviews in the study.

Table 1 lists the demographics of the entire sample; it should be noted that some variables (e.g., family structure, work status, youth race, and youth age) do not add up to 100% due to rounding error: 

Among the families with two-parent households, four had a parent/stepparent, ten had both biological parents, and one was a set of adopted parents. In five of the fifteen single-headed female households, the youths had regular interactions with the father. By extended families, there were two households with grandparents and one with an aunt. The median household size was four.

In terms of socioeconomic status, I did not ask specific income levels; instead, I determined their socio-economic status by asking about their jobs (part/full-time; occupation). Most of the families were poor or working class; all the families mentioned in the results section of this paper are poor or working class. The families primarily had Medicaid, with two other families mentioning they had insurance through the parent or legal guardian’s employment. 

As shown below in Table 2, most of the youths (30) had asthma, diabetes, or obesity. The other four had attention-deficit/hyperactivity disorder (ADHD), HIV, celiac disease, or a heart condition. The median time of managing their condition (as measured by the age of their diagnosis) was 11 years, with the range being from 2 months to 15 years.

In analyzing the data, my research assistants and I used Dedoose to code the interviews. We developed codes centered around family illness (e.g., past and present household/non-household), institutional involvements related to the youths’ health and other issues, past and present institutional involvements for other family members’ health or non-health issues, health information-seeking practices, medical technology, and views of doctors/medical staff. To ensure intercoder reliability, we initially all coded the same interview to ensure we had the same general understanding of the codes; then each interview had two coders. We also met regularly to discuss our coding to ensure consistency and to refine the codes as necessary. I analyzed the ethnographic fieldnotes on my own, using similar codes as in the interviews and writing memos related to family history of illness, institutional involvements, and views of medical staff. The rest of this paper presents both youth and parents’ perspectives, focusing mainly on the latter who were primarily mothers as they were more likely to be aware of and managing other family illnesses and other institutional involvements.

## 3. Results

**Figure 3:**
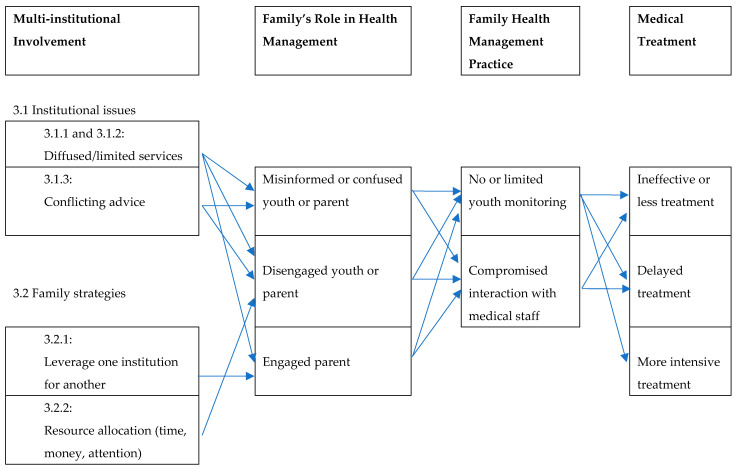
Pathways of multi-institutional involvement’s influence on youths’ medical treatment.

This section discusses the findings related to families’ multi-institutional involvement and its influence on the youths’ health management practices. It focuses on two types of challenges related to multi-institutional involvement. The first stems from instances when various institutions treat one member’s illness. The second addresses how families’ involvement in many institutions for health or non-health issues affect their ability to deal with any one illness. Class and race affect both types of multi-institutional involvement as families often face more logistical challenges in navigating between those institutions, as well as more discrimination if they are poor and/or people of color. The overview of the findings is found in Figure 3:

### 3.1. Multiple Institutions Managing the Youths’ Illness

Multiple institutions can get involved with the same health issue, which can complicate family and youths’ health management practices if those institutions do not work with each other or with the family.

#### 3.1.1. Diffused Services across Institutions

While one might expect more institutional involvement would help bolster the families’ efforts, such as schools having on-site nurses or hospital staff to conduct basic exams and administer asthma treatments (e.g., pumps and machines), this study found that more institutions do not necessarily enhance the medical care for the youths. Consider the Hernandez family as they work with three institutions (e.g., school, hospital, clinic) to manage 16-year-old Gigi’s newly diagnosed diabetes (all names of people and institutions are pseudonyms for confidentiality purposes). After an initial period of getting insulin shots, Gigi now must watch her food intake, take daily medication (Metformin), and go to regular check-ups with the endocrinologist at Weingart hospital. Her family also relies on her school nurse and a health clinic affiliated with another hospital, St. Simons, across the street. While Gigi is comfortable and, in some ways, prefers to see the school nurse over the doctor in the health clinic (mainly so she does not miss class), she mentions the school’s need to have her family involved and the subsequent administrative obstacles in her being able to access the school-based services. She explains, “they [school staff] have to take blood before lunch. But Mom forgot to sign the papers and they can’t do it without her signing it. I think they were supposed to give it to me, but they didn’t want to because I probably would lose it which is probably true.” While this seems like an innocuous delay in paperwork, it does raise the issue of how well schools and hospitals can coordinate with each other and the families. Moreover, according to Gigi, the school staff does not trust her to make sure her 45-year-old mother, Sulia, signs the paperwork, not giving her the opportunity to assume responsibility for that task as part of her health management practices. At the same time, Sulia thinks they are taking the blood and automatically sending the information about Gigi’s sugar levels to the clinic and Weingart hospital every week (which they are not). This lack of coordination or clarity in the process leads Sulia not to take an active role in managing Gigi’s diabetes.

#### 3.1.2. Limited Treatment Accessed via Multiple Institutions

Multi-institutional involvement further affects a youth’s health if those institutions have a “distorted engagement” [2] with the families, in which they provide the mandated services while maintaining suspicion about the family’s eligibility or deservedness for services. As such, these institutions’ services often are and feel to the families to be of lower quality when compared to institutions with primarily middle- and upper-class families who are seen more as clients or customers who can demand better service. Consider how Elisa, a 14-year-old Latina, and her 35-year-old mother, Norma, discuss Elisa’s counseling for oppositional defiant disorder (ODD) which she receives through Medicaid; she also has asthma and attention-deficit/hyperactivity disorder (ADHD). Elisa’s ODD affects her ability to manage her asthma; earlier in the interview, she mentions her ODD makes it hard to control her anger which leads to her yelling and fighting, both of which lead to her not being able to breathe well, triggering her asthma symptoms:

Elisa: I always get new teachers, uh not teachers, students. Like, students, they are only there for a little bit because they are in training. They are not an actual person that works there that’s gonna be my therapist for long term.

Interviewer: Have you told them that you want a therapist that is going to be there for a long time?

Elisa: (nods yes)

Interviewer: And what have they told you?

Elisa: (shakes head) …they would be giving you a student who lasts for a month, and then the next like a month or two and then the next month would be a different person so I can’t have a relationship. Like a relationship that I know I can trust that person. So, it’s hard to tell how I really feel if they keep on changing the person…

Interviewer: How many different people have you spoken to?

Elisa: More than ten … I’ve only had therapy for two years… they say the same thing, but then when I start on them, it just goes away.

Interviewer: Oh, so once you start getting comfortable, they switch them on you. And you’ve spoken to somebody in charge about that?

Elisa: (Shakes head yes)

Interviewer: And they don’t tell you anything like we’ll see…

Elisa: “We’ll see what we can do.” But that never happens.

If Elisa had more effective counseling to control her ODD, that would help improve her health outcomes related to her asthma. Instead, she finds herself having to talk to a constant rotation of therapists in training (more than ten in the two years she started this counseling) where it is “hard to tell how I really feel.” While she asked the organization for a more long-term therapist with whom she could build trust and rapport, it acknowledges her request but has not done anything about it for the two years she has been in counseling. This institutional constraint is affecting her ability to manage her multiple health conditions, yet it gets overlooked by others, including her mother, Norma, who attributes her daughter’s health issues as stemming from her being a “fighter”:

Norma: She’s a fighter… she don’t care… she’ll beat anybody that’s in her way… She don’t like you (fist hitting palm) that’s my little angel

Interviewer: Does she go to therapy?

Norma: She’s supposed to go to therapy, but she cursed them out so soon…there’s no way they’re gonna take her…

Interviewer: So, when was the last time she went to the doctors

Norma: One month ago, and then they closed the case, and they have to reopen it…In order to receive the medication from the psychiatrist she has to go to therapy

Interviewer: Oh, I see… so you have to reopen the case

Norma: I have to do everything… I have to fight …

Interviewer: When she went to therapy how long was the session

Norma: 45 min or less… she can have a bad day at school, and I go over there, and the therapist will say “what happened” or “your mother is with you, ok … it’s not bad.”

It is not clear if Norma knows about the constant rotation of student therapists. But even if she did, Norma does not appear to have other options to seek alternative therapy placements that might be more consistent or with more experienced counselors. She also has to fight to get her case reopened to be seen by the same counselors who were not helping her before. Norma recalls the therapist saying “it’s not bad” to Elisa, instead of helping her process her “bad day at school.” As a result, Norma is not able to help her daughter manage her ODD or asthma beyond continuing to advocate for her to get the current treatment that is ineffective.

#### 3.1.3. Conflicting Medical Advice across Institutions

Multi-sited health care can also complicate matters beyond just paperwork, especially when the youth receives conflicting advice about recommended medical treatment. Bob, a 16-year-old Latino, describes an experience where the school doctor and his hospital doctor had differing opinions about his asthma:

Bob: When I get sick, it hits me hard. So, I went to the doctor at my school … She told me something, and then when I went to my doctor [at the hospital], Flores—I love Flores—she was like, “What she said is not true.” [Laughs] … it was just like I felt the tension. … she [at school] was like, “Oh, do you have asthma?” I was like, “Yeah, I have asthma.” And she’s like, “Oh, you mighta had asthma attack.” I’m like, “I didn’t drop on the floor. I’m great. I’m alive.” She was like, “Oh, there’s different levels of asthma attacks. You can cough a lot; that’s an asthma attack. You can go on the ground; that’s the extreme.” … she’s [Dr. Flores] like, “That’s not an asthma attack. You did not have an asthma attack. Then my mom [said]… “Yes, you did have an asthma attack.”

Author: So, when you were at school … what happened? Did she send you home? Or did she send you to the doctor, the hospital?...

Bob: It was either a pill or some Motrin she gave me…. she gave me a pump, too. ‘Cause I never carry my pump. So, she’s like, “Here, a pump.” And then I had to take some pumps in front of her, ‘cause I forgot how to take pumps…

Author: And that helped you, the pumps, or no?

Bob: Not that I know of.

While it is common to expect different diagnoses from doctors without a clear idea of who to follow, what is analytically of note here is how Bob understands what happened and his role in managing his asthma. His mom and school doctor both thought it was an asthma attack; Bob and Dr. Flores, the hospital doctor, thought otherwise. While Bob does comply with the school doctor to take Motrin and the asthma pump, he also does not say he is going to change how he manages his own asthma. Rather, he ends by saying “I never carry my pump” and that he “forgot how to take pumps”. He also does not appear to feel compelled to start using his pump because in his view, the pump did not help in that situation. Moreover, he relies on his mother to decide when to go see Dr. Flores. Earlier in his interview, he mentions that he has been having a persistent bad cough for a month which may be related to his asthma or allergies or something else. Another hospital doctor, Dr. Peters, gave him steroids and said to come back in a month if it did not improve. Even though his condition has not improved, Bob looks to his mom to decide what to do. He says, “I follow her. Whatever she says. If she’s like, “Cough medicine for a whole month”, I’m like, “All right, let’s go. Let’s do it”. If she’s like, “Let’s go to the doctor”, I’m like, “Let’s go to the doctor”. Having multiple institutions and actors involved in Bob’s health complicates his ability to take more ownership of managing his asthma in two ways. One, the school doctor does not affirm his view of his symptoms when discussing his treatment. Two, both doctors and his mother’s actions appear to drive his care, either by proscribing the treatment plan (e.g., watching him take the pump) or deciding when to go to the doctor. Both ways allow him to remain passive, in the sense of waiting for these adults to either tell him what to do and in not seeking out more knowledge about his condition on his own.

### 3.2. Multiple Involvement across Issues/Members

Families’ dealings with various agencies for their members’ health and non-health issues influences their ability to engage in their youths’ health management practices. This section analyzes how that kind of involvement can add stress on families’ resources and affect existing levels of conflict, support, or cohesion in the family [28]; it also shows the implications for the kind of health care that the youth might receive.

#### 3.2.1. Leveraging Treatment via a Non-Health Institution

Families turn to non-medical institutions in certain situations to get access to and funding for medical treatment, such as parents advocating through the school system to provide certain types of therapy for their children with special needs [29]. For poor and minority families, this process can become complicated as they do not have the same levels of influence with the institutional staff as middle-class and white families. Consider the experience of Penny, a 41-year-old white mother of three children, as she tries to get her younger daughter on their father’s private insurance plan instead her current insurance through Medicaid:

Penny: They’re under Medicaid right now… I put in for court papers for child support for my seven-year-old, but they still haven’t done that yet. Once they go through with that process, she’ll probably be put on his [her father’s] insurance as well…He works for the police department, and he has HIP through the job …that’s what Ivy [her oldest child] has. Her basic insurance is HIP, and her secondary is Medicaid… If HIP doesn’t cover for something, her secondary insurance, which is Medicaid, covers. Like mostly for prescriptions—what do you call that?—referrals, Medicaid takes over for that.

Whether it is accurate or not, Penny believes that she needs to go through a non-health institution, Family Court, to get child support which then would enable her to get the dad to add his other daughter to his insurance which covers more than just Medicaid only (Penny’s youngest child, her son, does not have the same father as her two daughters which explains why she is not seeking to add him onto their father’s insurance). Her understanding is informed by her past experience with her oldest daughter, Ivy, who is on her dad’s insurance and for whom Penny gets child support. She feels compelled to go to court as the father does not do much for either daughter: she says, “He has multiple kids all over… my two girls are his, and he doesn’t do anything for them… but he collects tax money for her [Ivy].” This last quotation highlights the ways that poorer families are further disadvantaged in situations where the parents are no longer together and their already limited economic resources are further spread out across households. While the father appears to have a middle-class job in law enforcement, he is not helping to support his two daughters, besides paying child support and health care premiums for his oldest daughter. Meanwhile Penny is not working and must manage with less health care coverage for her two younger children who do not have the same comprehensive insurance that Ivy does. Penny is also dealing with Ivy’s care for asthma, allergies, ADHD, depression, and anxiety for which Ivy is seeing a medical doctor, a psychiatrist, and counselor. Furthermore, Penny is fighting with the school system to get an individualized education plan (IEP) that offers more services for her two youngest children who she suspects have ADHD and ADD. While she had provided a letter to request that evaluation in September, it still had not yet happened in February; she said she found out only in December that the school had lost that letter, so she had to redo it. Penny’s time and attention are being divided among several institutions (e.g., school system, court, two types of health insurance with different levels of coverage for her children), which prolongs and complicates her efforts to help manage her children’s medical issues.

#### 3.2.2. Limited Family Resources Spread over Multiple Cases

Multi-institutional involvement can affect parents’ involvement in their youth’s health management due to practical issues that then shape how medical staff view the family. Going back to the Hernandez family from the last section, Gigi explains why her mom, Sulia, has not signed the papers for her school to take her blood and send the information to the clinic or hospital: “So she has to go to the school [to get the form], but I guess she doesn’t have time because like there’s other kids, there’s my little sister and brother, so it’s very complicated.” Both Gigi’s younger brother and sister have their own medical and educational issues that require Sulia’s attention. Just dealing with the educational issues requires a lot of time as Gigi and her two siblings attend three different schools across the city, with Gigi’s school being the furthest from the house (at least one hour on the subway). Moreover, Sulia’s family does not always have the money to pay for those multiple trips on public transportation for her to shuttle back and forth between the schools (at the time of the fieldwork, a one-way subway fare was $2.75, and a monthly unlimited pass was $121). In addition, Sulia’s family is currently involved with five other institutions (e.g., Family Court, Supplemental Security Income) related to child support, public assistance, food stamps, disability benefits, and mental health treatment for its various members. Medical staff may not be fully aware of that multi-institutional involvement, potentially leading them to misconstrue the parents’ actions as ineffective or not caring about the youths’ medical condition. When I talk with Dr. Peters, Gigi’s doctor, at the clinic affiliated with St. Simons hospital about her diabetes in May 2016, he says he has not seen her for several months, when he says they should be making bimonthly appointments with the endocrinologist at Weingart hospital and regular follow-up visits with him. He attributes their noncompliance with that schedule to an adjustment period of a new diagnosis. But it could be more due to Sulia’s management of multiple institutional involvements and multiple illnesses at that time. She must deal with two school clinics, Dr. Peters’ clinic, and three hospitals to manage the other family members’ illnesses (e.g., her son has a serious ear infection and seizures; her mom had foot problems requiring hospitalization; and her oldest daughter recently gave birth to a baby which has ongoing breathing problems). Given Sulia’s attention being elsewhere, Gigi does not return to see Dr. Peters until nine months later, when I capture this conversation between the three of them in March 2017 at the clinic:

Dr. Peters: So, she needs a workup. She’s never had good control. Her sugars have always been high. When she was diagnosed, her A1C was like… 8.6. When we checked a year later, it was 8.8 And no one’s checked since. I just checked with them [Weingart hospital]. They haven’t seen her in the system for a year…. And the last time I had labs on you is May of last year… if it was me and my license, I wouldn’t just give you more meds. Your regimen right now isn’t working… We never had you in a good place… you still have to get to a good place.

Gigi: Yeah

Sulia: That’s why I said I run over there until they [Weingart] will see her… Because I know they, they, at school they do the, the-… you know? For her sugar.

Dr. Peters: Yeah. Do you want to try to make her a pediatric endocrine appointment down at St. Simons?

Sulia: We tried that… They say they don’t have all the stuff that she’s probably going to need.

Dr. Peters: They sent you up to Weingart.

Sulia: To Weingart.

Dr. Peters: Okay. So, we are going to do two things. We’re going to call them first thing tomorrow to make an appointment. And what, what’s your number? Because I’m going to call them tomorrow first thing to make an appointment. I’m just going to make it… As soon as I can. Even if it’s a month out. I know it sucks. But I want her to go.

In that last sentence, Dr. Peters implies he is the only one who wants her to go to Weingart and get her diabetes under control when, in fact, it was the hospital associated with his clinic, St. Simons, that delayed this process. The previous July, Dr. Peters sent Gigi to St. Simons to get her sugar levels tested, as it is closer to her house than Weingart. However, at that appointment, St. Simons’ staff did not do any of the required tests, saying she needed instead to go to Weingart (which is what Sulia means when she says “we tried that”). Meanwhile Sulia believes that the school is doing the daily monitoring of Gigi’s sugar and sending the reports to Dr. Peters and Weingart, which does not appear to be the case. So, while the onus is on the family to manage Gigi’s care across institutional settings, those institutions created some confusion as to which is addressing the different aspects of Gigi’s diabetes management. After this conversation with Dr. Peters, Gigi goes to Weingart and has to resume daily insulin shots, in addition to the medication. She is supposed to have a follow-up appointment the next month, which Sulia cancels due to a home emergency. The point here is not to suggest that the Hernandez family is irresponsible in managing Gigi’s diabetes; rather it is to show how the responsibility for that management gets distributed and refracted across institutional contexts and complicated by the family’s other illnesses and institutional involvements.

Similarly, dealing with institutions related to her own health and other family members’ health issues leads Lita, a 60-year-old Latina, to take a less active role in managing the health of her adopted daughter, Isabel. Lita is technically her aunt, as Isabel is her husband’s niece. Isabel was diagnosed as obese last year and suffering from polycystic ovary syndrome (PCOS) which can lead to possible infertility. While Lita does take her to medical appointments, the doctors’ main advice is for Isabel to lose weight to start getting her periods again. In describing how she helps Isabel in that regard, Lita says, “I was trying to put her on a diet but she don’t like to eat no vegetables. The only vegetable she eats is broccoli and it’s sometimes, not all the time and corn.” When I later ask in the interview if they ever disagree about how to handle Isabel’s weight, she says, “we start arguing and we keep going on and I get frustrated and I say, “You know what, leave it alone, go to your room, go watch TV, go—I don’t know what you want to do. Go clean your room. Leave me alone.” This half-hearted effort by Lita regarding Isabel’s health management could be attributed to the strain of her previous and current efforts in taking care of other family members’ and her own health issues. Lita has asthma and diabetes, as well as a history of anxiety and depression; she also says she has carpal tunnel syndrome and a herniated disk. In the past, Lita moved her whole family into her mother’s apartment several years ago to take care of her mother until she passed away; at the same time, she managed the care of her older daughter who died of cancer ten years ago, as well as the care of Isabel’s 11-year-old brother, Peter, who was born deaf and had surgery when he was three years old to restore his hearing. Currently, she coordinates the care of her brother, Martin, who lives with her and is bedridden after getting shot thirty years ago. She manages the home health aides who come daily (including interacting with the agency to request new aides if necessary) and takes care of some daily tasks such as cooking his food, helping him to go to the bathroom, and sterilizing tools. Peter, meanwhile, has been diagnosed with ADHD and takes a daily pill every morning. Lita takes Peter twice a week to a center that offers tutoring and counseling; each trip takes at least three hours because of the lengthy and unreliable public bus service. While her husband, older son, and his girlfriend also live in the same apartment, Lita is the only one who deals with her own and other family members’ health issues. As we are waiting to see the doctor for Isabel’s obesity, Lita explains how these tasks in managing multiple family members’ issues are starting to affect her:

She tells me how much they all are frustrating/irritating her: Peter, Isabel, her husband. She says that on top of all that she has to deal with her brother, Martin. Martin is still in the home in Harlem and cannot speak because of a trachea tube. He is going to stay there, which is better for everyone because he would yell when he was irritated; she can still hear him screaming her name even though he is not in the house anymore. Isabel says she hopes Peter will take over Martin’s room and Lita says they cannot. Isabel explains to me that this is because Martin is an ACS [Administration for Children Services] case. I do not understand, and Isabel explains that Lita is his legal guardian. Lita also says the house is in his name, so they have to keep the room open for him for that reason as well.

Even though Lita gets a respite from dealing with Martin’s daily needs in the home, she “can still hear him screaming her name,” distracting her. She also has been dealing for several months with Peter’s tutoring, due to a change of provider in the facility that now wants to stop the service; if she does not find another option, the tutoring will end in about a month. Moreover, while it might be good for Isabel to have her own room (which she currently shares with Peter), Peter cannot move into Martin’s room because of the rules of two other institutions: ACS, Administration for Children Services (child welfare) and NYCHA, New York City Housing Authority (public housing) for which Martin has open cases. All of those institutional involvements lead her to be even less invested in Isabel who she feels should manage her own medication on her own. This leads to the following interaction with Isabel’s doctor:

Dr. Singh says they have a lot to talk about—her period, her food, and weight. The doctor asks Isabel want she wants to talk about first. Isabel just looks at her and says whatever… Lita starts talking about how she keeps reminding Isabel to take her pill, but she does not take it. She also talks about Peter not taking his pill—she had to remind him this morning before they left to make sure to take his pills. She does not let Isabel get a word in edgewise, nor does the doctor who does not interrupt her. She says she yells at her [to take the pills] … but nothing works. Isabel finally says at one point that Lita is a “liar.”

Instead of working with Isabel and the doctor to address how to better manage Isabel’s condition, Lita simply expresses how she does her part by reminding her to take the medication while Isabel does not. Isabel feels her mom is misrepresenting the situation; she tells me later during the visit that Lita does remind her to take her medication but often it is at night, when she feels it is too late to take it. Isabel insists she is not intentionally forgetting to take the medication; that is why she feels Lita is lying to the doctor. While this interaction looks simply like a typical parent–teen conflict, the reasons behind it are more complicated as they stem from the ways that Lita feels frustrated in managing her family’s past and present institutional involvements. The ultimate result for Isabel is that she still is not in control of her weight, as she has gained another five pounds since her last visit a month ago; the doctor says she is exhibiting signs of pre-diabetes and they may consider surgery as the next step. 

## 4. Discussion

This paper has found that families’ involvement in youths’ health management practices are affected by their involvement in other institutions. We assume that it would be easier to access medical care if we increase the number of forums that provide it (e.g., hospitals, clinics, and schools). Yet, as shown in this paper, that expansion could lead to diffused treatment or conflicting medical advice. That, in turn, could confuse youths and parents further as to their respective roles in managing youths’ illnesses. This finding informs the research on chronic illness in two ways. One, families’ multi-institutional involvement related to the youths’ medical care complicates the process by which adolescents learn to manage their own illness on their own, as parents and youths have to keep track of who is doing what task for which institution. Institutions then play a key role in how parents and youths negotiate that division of labor. Secondly, it expands Corbin and Strauss’s [9] three interrelated types of work to manage a person’s illness, specifically pertaining to the illness work (practices related to the illness) and everyday life work (incorporating illness practices within other daily routines). Beyond the fact that the medical aspects of illness work are now spread across multiple institutions, families’ ability to manage that illness work is further affected by their everyday life work, which includes other institutional involvements for non-health issues in their family. 

In addition, the paper’s findings have provided insight into how family multi-institutional involvement exacerbates health disparities in more nuanced ways than found in previous work looking at the relationship between social class and health through fundamental cause theory [30,31], the effect of stress on family life [32], and co-morbidity [33,34,35,36,37,38,39]. Poor and minority families are often forced to engage with these institutions which offer lower quality services as compared to private institutions to which middle class and white parents have the ability and resources to access. Moreover, poor and minority families have little influence in how their cases in those institutions proceed. While they are actively advocating for their youths just like their middle-class and white counterparts, they are doing so in institutions that view them with skepticism. So multi-institutional involvement shapes families’ strategies in navigating the medical system, not only in the sense that they might have a different cultural health capital than middle-class families interacting with medical professionals, but also in how those medical staff view them as less credible or serious about their youths’ medical issues. That is, the staff could misconstrue the families’ actions related to the youths’ illness as misinformed or irresponsible when in fact those actions were due in large part to the families’ time, attention, and resources being spread out over those multiple institutions. In the end, the youths face delayed or lower quality treatment, and families perhaps are disengaged in managing the youths’ illness. These institutional processes add to health disparities both in the interactions between the families and medical staff and in the ways that staff may or may not know about the families’ involvements across institutions.

A commonly proposed idea to address the challenges raised by multi-institutional involvement is to improve coordination among institutions. However, that synergy could have the unintended effect of drawing families deeper into a multi-institutional maze [1] that could lead to greater surveillance of parents and youths. For example, punitive institutions could get involved and for longer periods of time, particularly in poor and minority families, such as the police who are called for mental health related incidents, or child welfare agencies [40] called in by the hospitals or schools to investigate the family for possible neglect. Yet at the same time, some families in my study reported that having the justice system (e.g., courts and police) or child welfare facilitate youths’ physical or mental health care was helpful. This paper has shown the ways that this uncertain outcome is shaped, at least in part, by the families’ involvement across institutions whose staff may or may not fully understand or even know of the extent of that involvement. The different pathways are depicted in Figure 3. Gigi’s family follows two pathways, diffused treatment and limited resources, that lead to a misinformed and disengaged parent which compromises their interactions with the doctor and ultimately leads to delayed and more intensive medical treatment for her diabetes. Penny’s example starts with her trying to leverage one institution for another which requires her to be a more engaged parent; however, there is limited monitoring of her children’s conditions as she awaits the court and schools’ decisions which ultimately leads to delayed treatment for those conditions. These pathways, starting with the multi-institutional involvement, show how families’ health management practices are not only due to their own views or ideas but also as a response to and interaction with various medical and non-medical agencies.

## 5. Conclusions

This paper has focused on family multi-institutional involvement to show another mechanism by which health inequalities are reproduced. It has argued for the need to pay more attention to the institutional role in family management of youth illnesses—mainly in how it constrains versus facilitates, and how it leads to families being blamed versus supported by institutions who remain ignorant of or make their own errors related to the families’ situations. Each family has a distinct multi-institutional experience that can become salient or relevant at different moments in managing youths’ illnesses. We need to have a better understanding of families’ institutional involvements to be able to assist them to empower youths to take care of their health, rather than assuming they are not taking the disease seriously. Furthermore, paying more attention to family multi-institutional involvement helps inform our understanding more broadly about health disparities, given that poor and minority families are more likely to be involved in liminal institutions that often do not provide timely and effective treatments, which only furthers the overall social inequalities.

## Figures and Tables

**Table 1:** Population characteristics.

		Household (*n* = 33)	Parent (*n* = 34)	Youth (*n* = 34)
Family Structure				
	Single parent	45%		
	Two parent	45%		
	Extended family	9%		
Work Status				
	Not working		39%	
	Part-time		18%	
	Full-time		39%	
	Unknown		3%	
Gender				
	Male		6%	41%
	Female		94%	59%
Race				
	Black		26%	24%
	White		6%	0
	Latinx		65%	59%
	Other (e.g., multiracial)		3%	18%
Age				
	12			15%
	13			9%
	14			18%
	15			26%
	16			21%
	17			9%
	18			3%
	Median		44 years	15 years
	Range		24–60 years	12–18 years

**Table 2:** Illnesses by Gender.

Illness	Total	Gender	
		Male	Female
Obesity	10 (29%)	4 (29%)	6 (30%)
Asthma	17 (50%)	5 (36%)	12 (60%)
Diabetes	3 (9%)	2 (14%)	1 (5%)
Other	4 (12%)	3 (21%)	1 (5%)
Total	34	14	20

## Data Availability

The data are not publicly available due to confidentiality reasons.
